# Modified recipe to inhibit fruiting body formation for living fungal biomaterial manufacture

**DOI:** 10.1371/journal.pone.0209812

**Published:** 2019-05-13

**Authors:** Jinhui Chang, Po Lam Chan, Yichun Xie, Ka Lee Ma, Man Kit Cheung, Hoi Shan Kwan

**Affiliations:** School of Life Sciences, The Chinese University of Hong Kong, Shatin, New Territories, Hong Kong; Osmania University, INDIA

## Abstract

Living fungal mycelium with abolished ability to form fruiting bodies is a self-healing substance, which is particularly valuable for further engineering and development as materials sensing environmental changes and secreting signals. Suppression of fruiting body formation is also a useful tool for maintaining the stability of a mycelium-based material with ease and lower cost. The objective of this study was to provide a biochemical solution to regulate the fruiting body formation, which may replace heat killing of mycelium in practice. The concentrations of glycogen synthase kinase-3 (GSK-3) inhibitors, such as lithium chloride or CHIR99021 trihydrochloride, were found to directly correlate with the development of fruiting bodies in the mushroom forming fungi such as *Coprinopsis cinerea* and *Pleurotus djamor*. Sensitive windows to these inhibitors throughout the fungal life cycle were also identified. We suggest the inclusion of GSK-3 inhibitors in the cultivation recipes for inhibiting fruiting body formation and regulating mycelium growth. This is the first report of using a GSK-3 inhibitor to suppress fruiting body formation in living fungal mycelium-based materials. It provides an innovative strategy for easy, reliable, and low cost maintenance of materials containing living fungal mycelium.

## Introduction

The development of fungal mycelium-based materials has beed fast over the past decade. Mycelium is the vegetative structure of fungi and is mainly composed of natural polymers. Living mycelium-based materials have a wide range of applications due to their self-assembly, self-healing, environmentally responsive nature, along with their moldable and tunable properties during growth. Dried mycelium is nontoxic, fire-resistant, mold-resistant, water-resistant, and biodegradable. It is also a great thermal insulator on top of its strength, durability and other beneficial features [1–13]. Under proper circumstances, the mycelium of typical mushroom-forming fungi aggregates to form mushrooms, which are the fruiting bodies spreading spores[14]. While the fruiting bodies cause conformational changes of the mycelium-based materials, the spores can cause allergy and infection in the susceptible population. In current production of mycelium-based materials, moulded products are heated or treated with fungicide to kill the living cells to stop fruiting body formation[15]. Such rendered mycelium-based materials retain few of the benefits of living materials. Therefore, new approaches are needed to inhibit fruiting body formation while keeping the mycelium alive to produce living mycelium-based materials with desirable qualities.

Kinases mediate cellular and developmental responses to environmental and internal signals, and kinase cascades play crucial roles in many signaling transduction pathways [16,17]. Phosphorylation of protein kinases affects the activity, location, stability, conformation, and protein-protein interaction of kinases. One interesting and putatively central regulatory kinase is glycogen synthase kinase-3 (GSK-3). GSK-3 is a serine/ threonine kinase of the CMGC family that is highly conserved in eukaryotes. GSK-3 is activated by the constitutive phosphorylation at a C-terminal tyrosine residue, but inactivated by the regulatory phosphorylation at an N-terminal serine residue which causes a conformational change blocking the catalytic domain [18]. Protein kinase A (PKA), protein kinase B (PKB) and protein kinase C (PKC) can transiently inhibit GSK-3 in response to various external signals. In fungi, these kinases are essential growth regulators in response to environmental stimuli[19–21].

GSK-3 has attracted widespread attention as a critical therapeutic target whereby lithium chloride (LiCl) is an archetypal GSK-3 inhibitor. Lithium (Li) exerts its pharmacological effects on mood stabilization, neurogenesis, neurotrophicity, neuroprotection, anti-inflammation, among others [18]. Lithium compounds are also suggested to be added during cultivation of some edible mushrooms to produce food biofortified with lithium [22,23]. Recent evidence suggests that low, non-toxic concentrations of LiCl have anti-inflammatory effects [24]. CHIR99021 trihydrochloride is a highly selective GSK-3 inhibitor [25]. Cisplatin has been shown to activate GSK-3, which induces the phosphorylation of C-terminal tyrosine but reduces the phosphorylation of N-terminal serine [26].

Two mushroom species from the order *Agaricales* were used in this study. *Coprinopsis cinerea*, a model mushroom-forming fungus, belongs to the family Psathyrellaceae [27]. The typical life cycle of *C*. *cinerea*, which includes stages of basidiospores, mycelium, hyphal knots, initials, stage-1 and -2 primordia, and young and mature fruiting bodies, can be finished within two weeks under laboratory conditions [28]. *Pleurotus djamor*, also known as the pink mushroom or tropical oyster mushroom, belongs to the family Pleurotaceae and is appreciated as an edible and medicinal mushroom in many countries.

This study aimed to provide a biochemical approach to inhibit fruiting body formation from mycelium-based materals. We demonstrated that LiCl and CHIR99021 trihydrochloride inhibited fruiting body formation, whereas cisplatin accelerated fruiting body development.

## Materials and methods

### Strains and cultivation conditions

Two GSK-3 inhibitors, LiCl (99%, Sigma-Aldrich, USA) and CHIR99021 trihydrochloride (99.2%, Tocris, USA), and one GSK-3 activator, Cisplatin (99%, Newbio Pharm-tech, Wuhan, China) were tested in *C*. *cinerea*, strain #326 (*A43mut B43mut pab1-1*, a generous gift from Prof. Hajime Muraguchi at Akita Prefectural University, Japan). LiCl was also tested in *P*. *djamor* (a generous gift from Prof. Yang Xiao at Huazhong Agricultural University, China). The *C*. *cinerea* strain used for gene expression level determination was also strain #326 since the transcriptomes of different developmental stages are available for this strain[29].

Mycelia of the above strains were stored at 4°C. A small agar piece from stock plates was then inoculated in freshly made agar plates for pre-culture. The pre-culture condition for *C*. *cinerea* was at 37°C in the dark on yeast extract-malt extract-glucose (YMG) agar (4 g yeast extract, 10 g malt extract, 4 g glucose and 10 g agar per litre) [30] in 9 cm petri dishes, while that for *P*. *djamor* was at 28°C in the dark on Potato Dextrose Agar (PDA, BD Difco) in 6 cm petri dishes. In each assay, a small agar piece with mycelium (0.8 cm diameter) from a 5-day-old pre-culture was inoculated in the middle of freshly made agar plates. *C*. *cinerea* was firstly cultured at 37 ^o^C in the dark until mycelia grew over the whole agar surface, then transferred to 25 ^o^C under a 12hours light /12hours dark cycle to induce fruiting body formation. *P*. *djamor was* cultivated at 28 ^o^C in the dark until mycelia occupied the whole agar surface, and transferred to 25 ^o^C under a 12hours light /12hours dark cycle. Triplicates were employed in each setup. Each 9 cm petri dish contained 34 g (±1 g) medium, and each 6 cm petri dish contained 10 g (±1 g) medium to standardize the nutrients and inhibitor/activator concentrations.

### Effect of GSK-3 inhibitors and activator

Three methods, varying in time and position, were tested to deliver LiCl. One method was to mix 1.5 g/L, 2 g/L (for *P*. *djamor)*, 3 g/L or 6g/L LiCl in the medium before autoclave sterilization, and the other methods were either to spread 1 mL sterilized LiCl solution (52.5 g/L, 105 g/L, 210 g/L) on the surface of agar before inoculation, or to add 1 mL sterilized LiCl solution (52.5 g/L, 105 g/L, 210 g/L) under the agar after the mycelia reached the edge of the petri dish. Adding 1 ml LiCl to 34 g agar made the final concentrations were approximate to the mixing methods. CHIR99021 trihydrochloride and cisplatin are not suggested to be autoclaved so 0.2-micron filters were used to remove bacteria in the solution. Then 1 mL CHIR99021 trihydrochloride solution (1 μM, 100 μM, 500 μM) or 1 mL saturated cisplatin solution (25 ^o^C) was spread on the surface of agar evenly before inoculation.

### Mycelial growth area determination

To examine the effect of LiCl on mycelial growth, LiCl powder was mixed in the YMG medium (0, 1.5 g/L and 3 g/L) before autoclave sterilization. Mycelial growth was recorded daily by marking the edge of the colonies on the plate bottom for six days after inoculating a *C*. *cinerea* pre-culture plug of 0.8 cm diameter. Three replicates were measured for each setup. Digital photos of the plate bottom with marks were taken, with a ruler in the same plane as the plates. The area occupied by mycelium was calculated using the Polygon Tool in the Analyzing Digital Images (ADI) software (https://www.umassk12.net/adi/).

### Sensitive windows to LiCl

The effects of LiCl at different developmental stages of *C*. *cinerea* were tested to find the sensitive windows. Agar piece with mycelium was inoculated on the center of a cellophane sheet placed on a YMG agar plate [29]. One mL of 105 g/L LiCl solution or 1 mL water was added between the cellophane sheet and the agar surface at the stages of: initial, stage-1primordium, stage-2 primordium, and young fruiting body. The growth status was recorded till three days after the control group formed mature fruiting bodies.

### Expression levels of GSK-3 target genes

The GSK-3 substrates were predicted by OrthoMCL V2.0.6 [31] with the default parameters (MCL inflation = 1.5; blastp *E*-value = 1e-5). A total of 83 GSK-3 target proteins reported in human and mouse were compared to the *C*. *cinerea* proteins, and 52 orthologues were identified (S1 Table). Among them, glycogen synthase (GS, CC1G_01973), eukaryotic translation initiation factor 1 (eIF1, CC1G_03881), and eukaryotic translation initiation factor eIF2 gamma subunit (eIF2-gamma, CC1G_09429) were picked for real-time PCR analysis, which also included GSK-3 (CC1G_03802) itself. Sequences of the primers used are listed in S2 Table.

To examine the effect of LiCl on the expression levels of target genes of GSK-3, 1 mL water or LiCl solution (52.5 g/L and 105 g/L, equivalent to 1.5 g/L and 3 g/L in previous sections) was spread on the surface of agar and then covered by a cellophane sheet for easier harvest of the mycelium. Mycelium from *C*. *cinerea* strain #326 was inoculated on top of the cellophane sheet. Three biological replicates were employed for each setup. After a 4-day incubation at 37°C in the dark, total RNAs were extracted using RNeasy Plant Mini Kit (Qiagen). The RNA concentration was measured by a NanoDrop Spectrophotometers (Thermo Scientific). RNA products (500ng) were used to synthesize cDNA using iScript gDNA clear cDNA Synthesis Kit (Bio-Rad). Quantitative real-time PCR (qPCR) was performed with three technical replicates on an Applied Biosystems 7500 Real-Time PCR system using SsoAdvanced universal SYBR Green Supermix (Bio-Rad) according to the standard protocol: 1 cycle at 95°C for 30 seconds and 40 cycles at 95°C for 15 seconds, annealing at 60°C for 60 seconds. Beta-tubulin was used as an endogenous control for normalization. Negative control was employed for each primer pair to eliminate false positive results.

## Results

### GSK-3 inhibitors and activator affect fruiting body development

**Fig 1:**
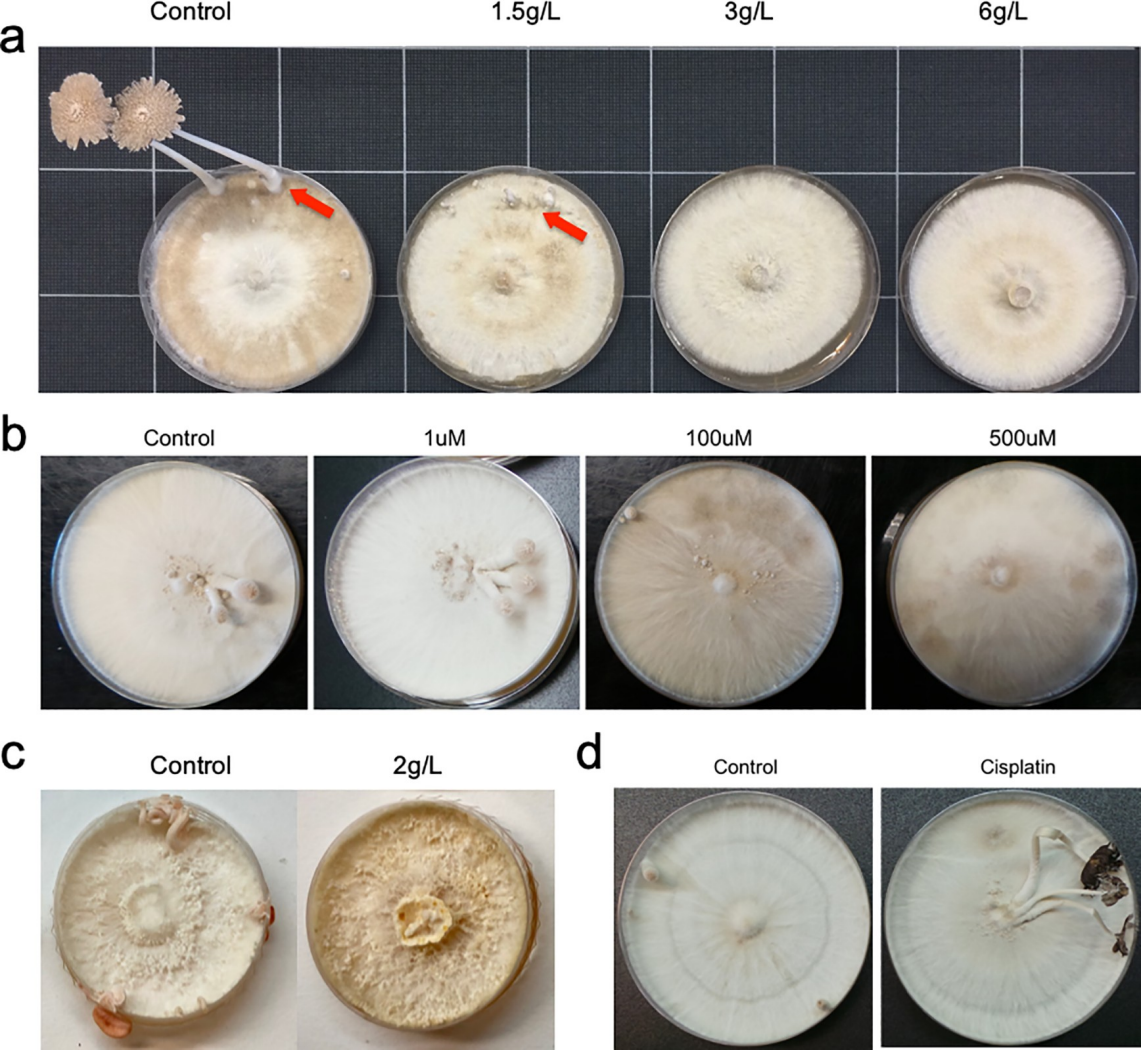
Effect of GSK-3 inhibitors and activator on the fruiting body development. (a) LiCl had inhibitory effect on the fruiting body development of *C*. *cinerea* in a concentration-dependent manner. Mature fruiting bodies were produced in the control group, while the 1.5g/L LiCl treated group produced only initials and primordia. No initiation was observed in the groups treated with higher concentrations of LiCl, in the following 30 days. (b) CHIR99021 trihydrochloride had inhibitory effect on the fruiting body development of *C*. *cinerea* in a concentration-dependent manner. Young fruiting bodies developed in the control group and 1 μM treated group, while only stage-1 primordia were developed on plates treated with 100 μM. Mycelia on plates treated with 500 μM remained in the mycelium stage in the following 30 days. (c) LiCl inhibited the fruiting body development of *P*. *djamor*. Mature fruiting bodies were produced in the control group, while the 2g/L LiCl treated group remained in the mycelium stage in the following 30 days. (d) Cisplatin accelerated the fruiting body development of *C*. *cinerea*. The plates treated with 1 mL saturated cisplatin solution had mature fruiting bodies and began autolysis, while the control group only developed into stage-2 primordia.

As shown in Fig 1A, the effect of LiCl on *C*. *cinerea* fruiting body development was tested. While the control group developed into mature fruiting bodies, 1.5g/L LiCl treated group only developed into stage-1 primordia. The plates treated with 3g/L LiCl were arrested in the mycelium stage, and no initials or hyphal knots can be observed in the following 30 days. The mycelium treated with 6g/L LiCl stopped growing before reaching the edge of the petri dish. These results showed that LiCl of higher concentrations had stronger inhibitory effect on *C*. *cinerea* fruiting body development.

The three delivery methods of LiCl, either mixed in the agar, on the surface of agar, or under the agar, showed no differences and all efficiently inhibited the fruiting body development. LiCl is not sensitive to heat treatment and can be autoclaved to sterilize the solution. Any of these delivery methods can be chosen in large-scale manufacture of the materials.

One more selective GSK-3 inhibitor, CHIR99021 trihydrochloride, was tested on *C*. *cinerea*. As shown in Fig 1B, young fruiting bodies developed on the control plates treated with water and the plates with 1 μM CHIR99021 trihydrochloride. Stage-1 primordia were developed on the plates treated with 100 μM CHIR99021 trihydrochloride. Mycelia in the plates treated with 500 μM CHIR99021 trihydrochloride remained arrested in the mycelium stage in the following 30 days. These results showed an stronger inhibitory effect on *C*. *cinerea* fruiting body development by CHIR99021 trihydrochloride at higher concentrations.

To demonstrate that GSK-3 inhibitor is also important in fruiting body formation in other mushrooms, the effect of LiCl on *P*. *djamor* was tested (Fig 1C). LiCl was added to PDA medium before autoclave. While mature fruiting bodies developed on the control plates, the plates treated with 2g/L LiCl failed to develop fruiting bodies in the following 30 days. These results showed an inhibitory effect on *P*. *djamor* fruiting body development by LiCl.

With the aforementioned positive results that GSK-3 inhibitors can inhibit fruiting body development, we hypothesized that GSK-3 activity is associated with the fruiting body development. A GSK-3 activator, cisplatin, was then tested for its effect on *C*. *cinerea* fruiting body development (Fig 1D). The cisplatin treated group showed an accelerated development since the formation of hyphal knot, and the mature fruiting bodies appeared two days earlier than the control group, which only developed into young fruiting bodies. These results showed a promoting effect of cisplatin on *C*. *cinerea* fruiting body development.

These data support the observation that among fungal species of the order Agaricales, GSK-3 inhibitors inhibit fruiting body formation, whereas GSK-3 activator activates fruiting body formation.

### LiCl promotes mycelium growth

**Fig 2:**
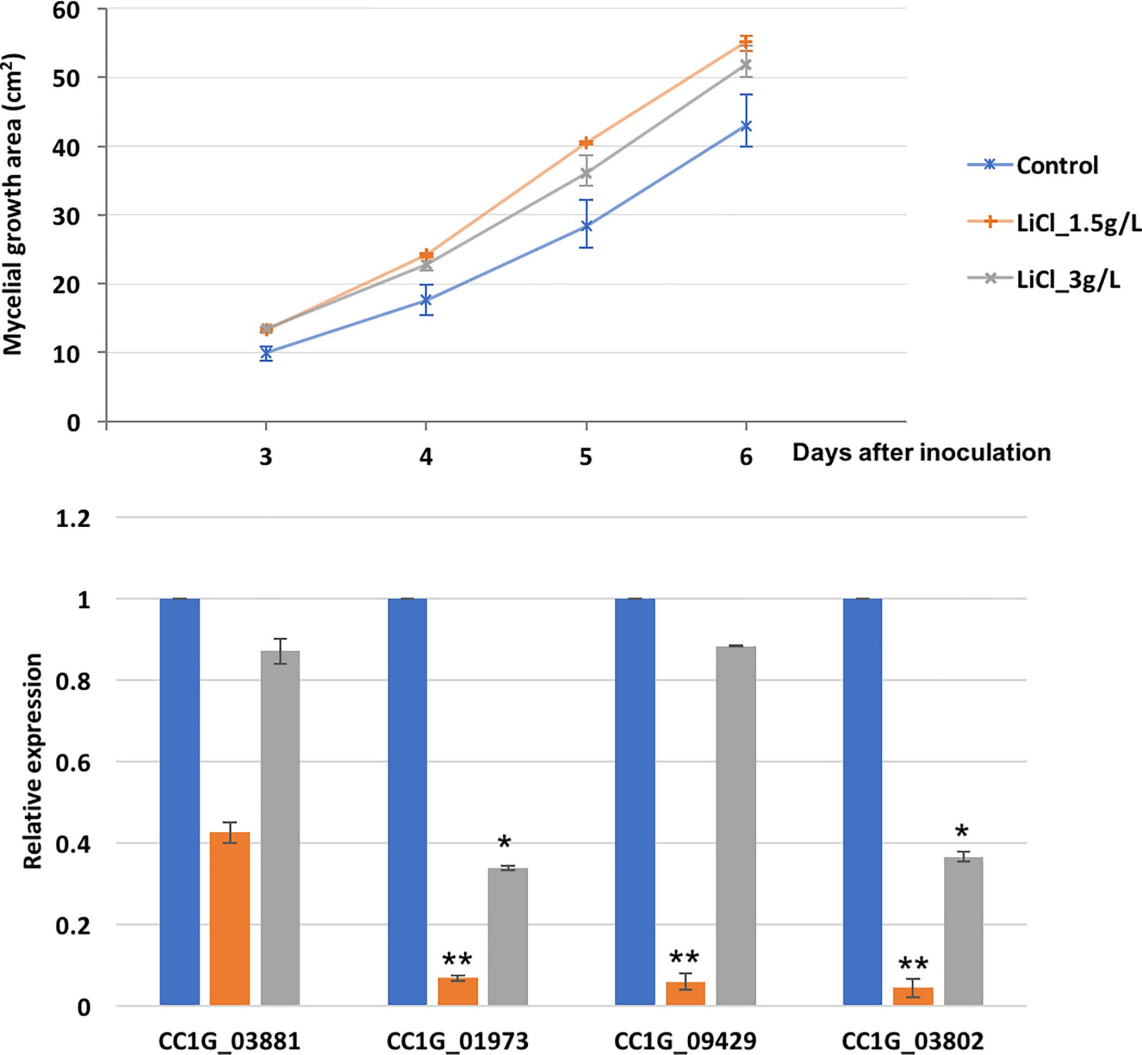
**(a) Mycelial growth of *C*. *cinerea* with different concentrations of LiCl**. The average mycelial growth area of inoculums on medium mixed with 1.5g/L were larger than that of the controls from day 3 to day 6, while the one with 3g/L LiCl was larger than that of the controls only on day 3 and day 5 (n = 3, p<0.05). The error bars encompass the lowest and highest values. **(b) Gene expression levels by real-time PCR**. The expression levels of GS (CC1G_01973), eIF1 (CC1G_03881), eIF2-gamma (CC1G_09429), and GSK-3 (CC1G_03802) decreased under LiCl treatment. Each value is expressed as mean ± SD (*P < 0.05 and **P < 0.001).

Mycelial growth area of the biological triplicates was recorded daily. Fig 2A shows the average mycelial growth area of each group with error bars showing the maximum and minimum values. As the inoculum usually needs time to adapt to a new environment and absorb nutrients, the mycelium grew only slowly in the first two days. Differences appeared on day 3 and day 4 after inoculation, with both LiCl treated groups growing faster than the control group (p < 0.05, one way Student's t test). On day 5 and day 6, the mycelium from 1.5 g/L LiCl treated group still grew faster than the control group (p < 0.05), while the 3 g/L LiCl treated group had little difference with the control group. The results showed that proper concentrations of LiCl would accelerate the mycelium growth.

This is an ideal property of GSK-3 inhibitor for large-scale production of mycelium-based material. The modified recipe with addition of a proper concentration of LiCl, can not only inhibit fruiting body formation but also speed up mycelium growth, and hence shorten the manufacture cycle and lower the cost.

### LiCl affects gene expression levels

To investigate the LiCl effect on gene expression in the mycelium stage, the expression levels of GSK-3 and its target genes were tested. As shown in Fig 2B, real-time PCR results showed that 1.5 g/L LiCl reduced the expression of GSK-3 itself (CC1G_03802), as well as GS (CC1G_01973), eIF1 (CC1G_03881), and eIF2-gamma (CC1G_09429) significantly (p <0.001, two-way Student's t test). 3 g/L LiCl reduced the expression of GS and GSK-3 (p <0.05). The change in gene expression supports that LiCl affects GSK-3 and its downstream genes.

### The stages before stage-1 primordium are sensitive to LiCl

**Fig 3:**
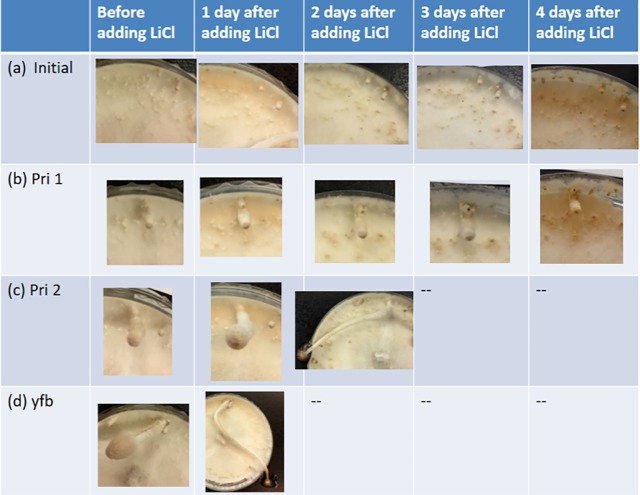
Development of *C*. *cinerea* treated with LiCl at different stages. Addition of LiCl at stages of initial and stage-1 primordium inhibited further development. Addition of LiCl at stages of stage-2 primordium and young fruiting body did not inhibit the fruiting body development.

The bottleneck to produce living mycelium-based material is to avoid the formation of fruiting bodies. Upon environmental stimuli including nutrient depletion, light/dark cycle, and cold shock, mycelia aggregate into hyphal knots, followed by fruiting body initials. Initials then develop into stage-1 and -2 primordia, young and eventually mature fruiting bodies. We explored all possible chances in existing production lines to introduce a GSK-3 inhibitor, specifically, LiCl, which is cheaper than the other GSK-3 inhibitors. The previous sections demonstrated that LiCl could be added from pre-inoculation to mycelium extension. Hyphal knot is a short stage that is difficult to define by naked eyes. So only stages after hyphal knot were tested. As shown in Fig 3, the addition of LiCl at stages of initial and stage-1 primordium led to the arrest of development. However, stage-2 primordium and young fruiting bodies continued to develop into mature fruiting bodies after LiCl treatment. So, the stages of mycelium, initial and stage-1 primordium are sensitive windows to LiCl.

## Discussion

This study has demonstrated that LiCl and CHIR99021 trihydrochloride treatments inhibited fruiting body initiation in a concentration-dependent manner (Fig 1A and 1B). In many instances, one would prefer for a living fungal mycelium to refrain from developing into fruiting bodies so that the mycelium could be easily maintained without concerns of loss in its shape, form, or consistency. Compared to the current method of heat-killing fungal mycelium to prevent fruiting body formation, a living version of mycelium that simply does not form fruiting bodies is far more desirable, considering its living nature and thus self-healing potential. Therefore, we suggest the inclusion of LiCl or CHIR99021 trihydrochloride in recipes to manufacture living fungal mycelium-based materials that exhibit controlled fruiting body development. In other cases, promoting fruiting body development may be of interest. For instance, when the intended goal is to produce as many fruiting bodies (*e*.*g*., mushrooms and truffles) as possible in a defined time period, having an enhanced fruiting body development would be beneficial. Although cisplatin could accelerate fruiting body formation, more studies about its safety and influence on human health are needed.

LiCl affected the expression levels of GSK-3 and three substrates. In a previous study of *C*. *cinerea* strain #326 [29], the expression level of eIF1 (CC1G_03881) remained stable from mycelium to primordium, but increased significantly after stage-2 primordium; and the expression levels of GS (CC1G_01973) and GSK-3 (CC1G_03802) increased during the development from mycelium to primordium, while eIF2-gamma (CC1G_09429) decreased (S1 Fig). GSK-3, GS and eIF1 may be essential to fruiting body development, but LiCl treatment reduces their expressions.

**Fig 4:**
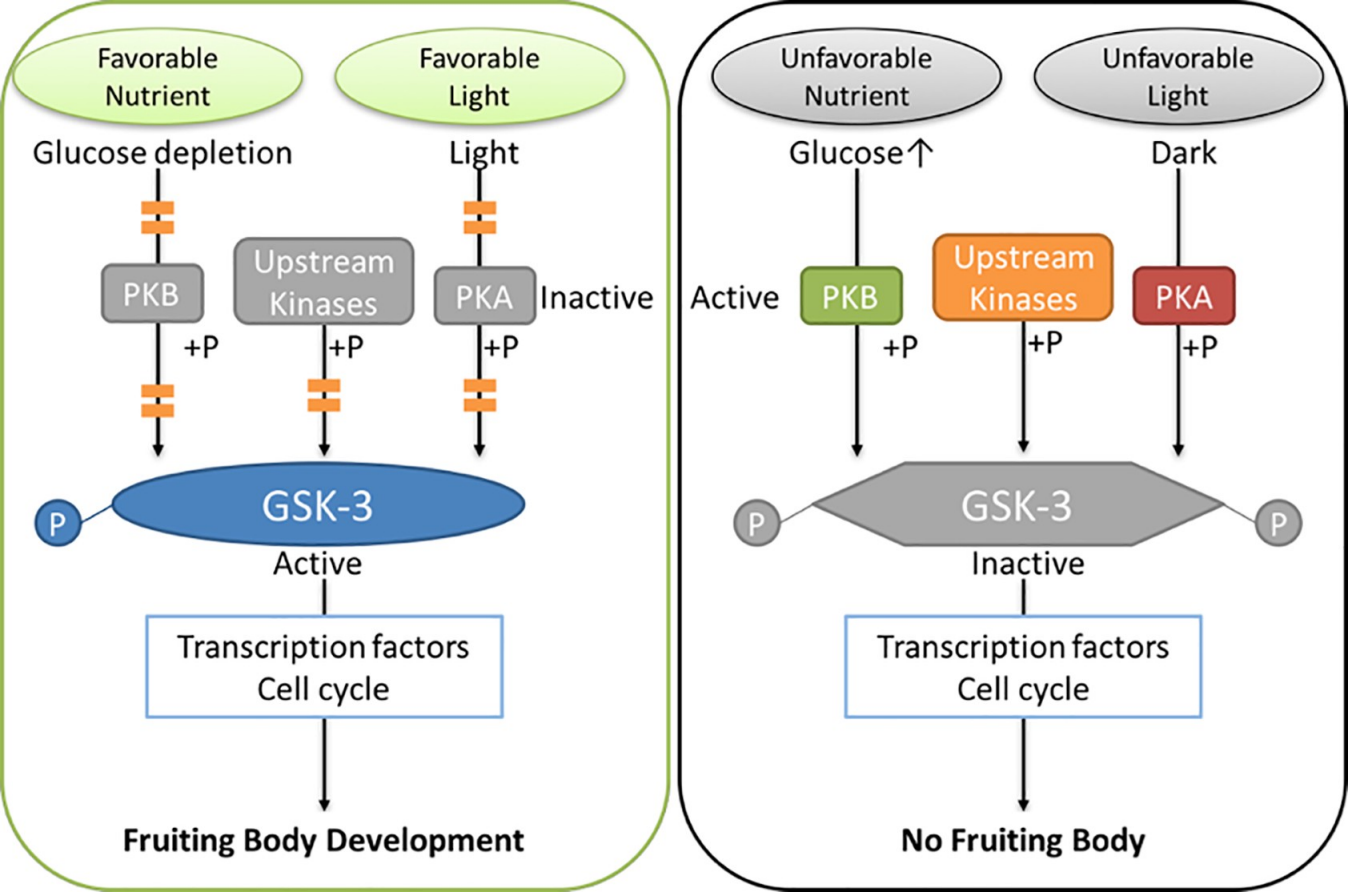
GSK-3 as a master-switch of fruiting body formation. GSK-3 could be the links between environmental stimuli and developmental responses, as a master-switch of fruiting body formation. While GSK-3 is constitutively active under favorable conditions, any unfavorable stimuli could inactivate it and turn off fruiting body development.

Given that both LiCl and CHIR99021 trihydrochloride are inhibitors to GSK-3, their effects of fruiting inhibition might be mediated through the inactivation of GSK-3. GSK-3 has important role in cell-fate specification, leading to cell differentiation or apoptosis or development through a number of signaling pathways[32–35]. We proposed that GSK-3 could be the links between environmental stimuli and developmental responses, as a master-switch of fruiting body formation (Fig 4). While GSK-3 is constitutively active under favorable conditions, any unfavorable stimuli could inactivate it and turn off the fruiting body development. The activity of GSK-3 may directly or indirectly determine fruiting body development.

When the GSK-3 activity is limited by inhibitors, undifferentiated cells are proliferative. GSK-3 inhibitors have been shown to maintain mouse and human embryonic stem cells in undifferentiated status, while removing the inhibitors promotes differentiation into multiple cell lineages [36]. Some transcription factors phosphorylated by GSK-3 will be targeted by ubiquitination for proteasome-mediated degradation. Substrates with less GSK-3 phosphorylation and ubiquitination may have prolonged half-lives to enhance cell proliferation [37]. Therefore, GSK-3 inhibitors, which functions like unfavorable environmental signals, may inhibit fruiting body formation through interfering the cell differentiation. Deeper studies are needed to discover the mechanisms in the future.

GSK-3 might be targeted for producing living fungal myceliums with an enhanced or inhibited fruiting body development profile, by either a permanent means (*e*.*g*., GSK-3 knockdown or GSK-3 knockout fungal strain) or a transient means (*e*.*g*., application of an activator or inhibitor of GSK-3 present in the medium for fungi). While the former may be easier to maintain in the long term, efforts involved in the initial stage of establishing the genetically modified fungal strains are tremendously more significant both in cost and in time. In contrast, the latter offers the benefits of flexibility and low-cost use, when the GSK-3 activator or inhibitor can be readily removed at an appropriate time so that the fungus may resume its normal life cycle of different phases.

The concentration range is narrow for lithium salts enhancing mycelium growth. A high concentration of LiCl may inhibit the mycelium growth, especially in *Trichoderma* species, which is a common contamination of the edible mushrooms [38]. Thus, while LiCl can be applied to prevent fruiting in some mushroom-forming fungi, it can also inhibit contamination during manufacture in some scenarios. In addition to LiCl, other agents that specifically target GSK-3 may also prevent the development of fruiting bodies. Other known GSK-3 inhibitors include: maleimide derivatives, staurosporine and organometallic inhibitors, indole derivatives, paullone derivatives, pyrazolamide derivatives, pyrimidine and furopyrimidine derivatives, oxadiazole derivatives, thiazole derivatives, and miscellaneous heterocyclic derivatives [18,39].

According to the sensitive windows to LiCl, a basic production pipeline is designed for adding GSK-3 inhibitors for producing living mycelium-based materials. A production pipeline may include all or part of the following procedures: 1) cultivation substrates mixing and autoclave; 2) inoculation; 3) mycelium 1st growth; 4) molding and pressurize; 5) mycelium 2nd growth; 6) pressurize (optional); 7) mycelium 3rd growth (optional); and 8) air-dry to finalized product. LiCl or other GSK-3 inhibitors can be added at any time from procedure 1) to 7), by mixing in cultivation substrate before autoclave, adding on the surface before inoculation, or spraying to the mycelium after a period of growth.

In conclusion, LiCl, CHIR99021 trihydrochloride or other GSK-3 inhibitors can be applied in the manufacture of mycelium-based materials, which can shorten the production cycle, reduce the cost for maintenance of mycelium materials, and therefore achieve a higher level of cost-effectiveness.

## Supporting information

S1 Fig The expression profiles of GSK-3 and three target genes at different developmental stages. The values of expression levels were extracted from previous transcriptome study.
(TIFF)

S1 Table Gene ID of GSK-3 substrates in *C. cinerea*. (PDF)

S2 Table Primer sequences for Real-time PCR. (PDF)

## References

[1] Jones M, Bhat T, Huynh T, Kandare E, Yuen R, Wang CH, et al Waste-derived low-cost mycelium composite construction materials with improved fire safety. Fire and Materials. 2018;42: 816–825. 10.1002/fam.2637

[2] Nguyen PQ, Courchesne NMD, Duraj-Thatte A, Praveschotinunt P, Joshi NS. Engineered Living Materials: Prospects and Challenges for Using Biological Systems to Direct the Assembly of Smart Materials. Advanced Materials. 2018 p. 1704847 10.1002/adma.201704847 29430725 PMC6309613

[3] Appels FVW, Dijksterhuis J, Lukasiewicz CE, Jansen KMB, Wösten HAB, Krijgsheld P. Hydrophobin gene deletion and environmental growth conditions impact mechanical properties of mycelium by affecting the density of the material. Scientific Reports. 2018;8 10.1038/s41598-018-23171-2 29549308 PMC5856774

[4] Camere S, Karana E. Fabricating materials from living organisms: An emerging design practice. JOURNAL OF CLEANER PRODUCTION. 2018;186: 570–584. 10.1016/j.jclepro.2018.03.081

[5] Khamrai M, Banerjee SL, Kundu PP. A sustainable production method of mycelium biomass using an isolated fungal strain Phanerochaete chrysosporium (accession no: KY593186): Its exploitation in wound healing patch formation. Biocatalysis and Agricultural Biotechnology. 2018;16: 548–557. 10.1016/j.bcab.2018.09.013

[6] Jones M, Bhat T, Huynh T, Kandare E, Yuen R, Wang CH, et al Waste-derived low-cost mycelium composite construction materials with improved fire safety. Fire and Materials. 2018 10.1002/fam.2661 30996511 PMC6463531

[7] Kilaru S, Hoegger PJ, Kües U. The laccase multi-gene family in Coprinopsis cinerea has seventeen different members that divide into two distinct subfamilies. Current Genetics. 2006;50: 45–60. 10.1007/s00294-006-0074-1 16775746

[8] Grimm D, Wösten HAB. Mushroom cultivation in the circular economy. Applied Microbiology and Biotechnology. 2018 pp. 7795–7803. 10.1007/s00253-018-9226-8 30027491 PMC6132538

[9] Appels FVW, Camere S, Montalti M, Karana E, Jansen KMB, Dijksterhuis J, et al Fabrication factors influencing mechanical, moisture- and water-related properties of mycelium-based composites. Materials & Design. 2018; 10.1016/J.MATDES.2018.11.027

[10] Silverman J. Development and testing of mycelium-based composite materials for shoe sole applications. 2018;

[11] Karana E, Blauwhoff D, Hultink E, Camere S. When the Material Grows: A Case Study on Designing (with) Mycelium-based Materials. 2018;12: 119–136.

[12] Nguyen PQ, Courchesne NMD, Duraj-Thatte A, Praveschotinunt P, Joshi NS. Engineered Living Materials: Prospects and Challenges for Using Biological Systems to Direct the Assembly of Smart Materials. Advanced Materials. 2018 10.1002/adma.201704847 29430725 PMC6309613

[13] Grimm D, Wösten HAB. Mushroom cultivation in the circular economy. Applied Microbiology and Biotechnology. 2018 pp. 7795–7803. 10.1007/s00253-018-9226-8 30027491 PMC6132538

[14] Kües U, Subba S, Yu Y, Sen M. Regulation of fruiting body development in Coprinopsis cinerea. 2016;

[15] Haneef M, Ceseracciu L, Canale C, Bayer IS, Heredia-Guerrero JA, Athanassiou A. Advanced Materials From Fungal Mycelium: Fabrication and Tuning of Physical Properties. Scientific Reports. Nature Publishing Group; 2017;7: 41292 10.1038/srep41292 28117421 PMC5259796

[16] Kosti I, Mandel-Gutfreund Y, Glaser F, Horwitz B. Comparative analysis of fungal protein kinases and associated domains. BMC Genomics. 2010;11: 133 10.1186/1471-2164-11-133 20178650 PMC2838846

[17] Wang C, Zhang S, Hou R, Zhao Z, Zheng Q, Xu Q, et al Functional analysis of the kinome of the wheat scab fungus Fusarium graminearum. Howlett BJ, editor. PLoS pathogens. Public Library of Science; 2011;7: e1002460 10.1371/journal.ppat.1002460 22216007 PMC3245316

[18] Takahashi-yanaga F. Activator or inhibitor? GSK-3 as a new drug target. Biochemical Pharmacology. Elsevier Inc.; 2013;86: 191–199. 10.1016/j.bcp.2013.04.022 23643839

[19] Andoh T, Hirata Y, Kikuchi A. Yeast glycogen synthase kinase 3 is involved in protein degradation in cooperation with Bul1, Bul2, and Rsp5. Molecular and cellular biology. 2000;20: 6712–20. 10958669 10.1128/mcb.20.18.6712-6720.2000 PMC86186

[20] Moore SF, van den Bosch MTJ, Hunter RW, Sakamoto K, Poole AW, Hers I. Dual regulation of glycogen synthase kinase 3 (GSK3)α/β by protein kinase C (PKC)α and Akt promotes thrombin-mediated integrin αIIbβ3 activation and granule secretion in platelets. The Journal of biological chemistry. 2013;288: 3918–28. 10.1074/jbc.M112.429936 23239877 PMC3567645

[21] Tataroğlu Ö, Lauinger L, Sancar G, Jakob K, Brunner M, Diernfellner ACR. Glycogen synthase kinase is a regulator of the circadian clock of Neurospora crassa. The Journal of biological chemistry. 2012;287: 36936–43. 10.1074/jbc.M112.396622 22955278 PMC3481296

[22] Mleczek M, Siwulski M, Rzymski P, Budzyńska S, Gąsecka M, Kalač P, et al Cultivation of mushrooms for production of food biofortified with lithium. European Food Research and Technology. 2017;243: 1097–1104. 10.1007/s00217-016-2823-9

[23] De Assunão LS, Da Luz JMR, Da Silva MDCS, Vieira PAF, Bazzolli DMS, Vanetti MCD, et al Enrichment of mushrooms: An interesting strategy for the acquisition of lithium. Food Chemistry. Elsevier; 2012;134: 1123–1127. 10.1016/j.foodchem.2012.03.044 23107736

[24] Zhang F, Phiel CJ, Spece L, Gurvich N, Klein PS. Inhibitory phosphorylation of glycogen synthase kinase-3 (GSK-3) in response to lithium. Evidence for autoregulation of GSK-3. The Journal of biological chemistry. American Society for Biochemistry and Molecular Biology; 2003;278: 33067–77. 10.1074/jbc.M212635200 12796505

[25] Ring DB, Johnson KW, Henriksen EJ, Nuss JM, Goff D, Kinnick TR, et al Selective glycogen synthase kinase 3 inhibitors potentiate insulin activation of glucose transport and utilization in vitro and in vivo. Diabetes. 2003;52: 588–95. 12606497 10.2337/diabetes.52.3.588

[26] Park H-J, Kim H-J, Bae G-S, Seo S-W, Kim D-Y, Jung W-S, et al Selective GSK-3β inhibitors attenuate the cisplatin-induced cytotoxicity of auditory cells. Hearing Research. Elsevier; 2009;257: 53–62. 10.1016/j.heares.2009.08.001 19666099

[27] Stajich JE, Wilke SK, Ahrén D, Hang C, Birren BW, Borodovsky M, et al Insights into evolution of multicellular fungi from the assembled chromosomes of the mushroom Coprinopsis cinerea (Coprinus cinereus). 10.1073/pnas.1003391107 20547848 PMC2900686

[28] Plaza D, Lin C-W, van der Velden NS, Aebi M, Künzler M, Dacks J, et al Comparative transcriptomics of the model mushroom Coprinopsis cinerea reveals tissue-specific armories and a conserved circuitry for sexual development. BMC Genomics. BioMed Central; 2014;15: 492 10.1186/1471-2164-15-492 24942908 PMC4082614

[29] Muraguchi H, Umezawa K, Niikura M, Yoshida M, Kozaki T, Ishii K, et al Strand-specific RNA-seq analyses of fruiting body development in Coprinopsis cinerea. PLoS ONE. Public Library of Science; 2015;10: e0141586 10.1371/journal.pone.0141586 26510163 PMC4624876

[30] Rao PS, Niederpruem DJ. Carbohydrate metabolism during morphogenesis of Coprinus lagopus (sensu Buller). Journal of bacteriology. 1969;100: 1222–8. 5391229 10.1128/jb.100.3.1222-1228.1969 PMC250298

[31] Li L, Stoeckert CJ, Roos DS. OrthoMCL: identification of ortholog groups for eukaryotic genomes. Genome research. Cold Spring Harbor Laboratory Press; 2003;13: 2178–89. 10.1101/gr.1224503 12952885 PMC403725

[32] Tataroglu O. Role of Glycogen Synthase Kinase (GSK) in temperature compensation of the. 2011; 1–88.

[33] Ninkovic J, Stigloher C, Lillesaar C, Bally-Cuif L. Gsk3beta/PKA and Gli1 regulate the maintenance of neural progenitors at the midbrain-hindbrain boundary in concert with E(Spl) factor activity. Development (Cambridge, England). 2008;135: 3137–3148. 10.1242/dev.020479 18725518

[34] Casas-Flores S, Rios-Momberg M, Rosales-Saavedra T, Martínez-Hernández P, Olmedo-Monfil V, Herrera-Estrella A. Cross Talk between a Fungal Blue-Light Perception System and the Cyclic AMP Signaling Pathway. Eukaryotic Cell. 2006;5: 499–506. 10.1128/EC.5.3.499-506.2006 16524905 PMC1398060

[35] Zanolli F, Magalhães R, Paula D, Carlos L, Barbosa B, Francisco H, et al cAMP signaling pathway controls glycogen metabolism in Neurospora crassa by regulating the glycogen synthase gene expression and phosphorylation. Fungal Genetics and Biology. Elsevier Inc.; 2010;47: 43–52. 10.1016/j.fgb.2009.10.011 19883780

[36] Kirby LA, Schott JT, Noble BL, Mendez DC, Caseley PS, Peterson SC, et al Glycogen synthase kinase 3 (GSK3) inhibitor, SB-216763, promotes pluripotency in mouse embryonic stem cells. Cooney AJ, editor. PloS One. Public Library of Science; 2012;7: e39329 10.1371/journal.pone.0039329 22745733 PMC3383737

[37] Westermarck J. Regulation of transcription factor function by targeted protein degradation: an overview focusing on p53, c-Myc, and c-Jun Transcription Factors. Humana Press, Totowa, NJ; 2010 pp. 31–6. 10.1007/978-1-60761-738-9_2 20694659

[38] Wildman H. G. Lithium chloride as a selective inhibitor of Trichoderma species on soil isolation plates. Mycological Research. Elsevier; 1991;95: 1364–1368. 10.1016/S0953-7562(09)80386-9

[39] Selenica ML, Jensen HS, Larsen AK, Pedersen ML, Helboe L, Leist M, et al Efficacy of small-molecule glycogen synthase kinase-3 inhibitors in the postnatal rat model of tau hyperphosphorylation. British journal of pharmacology. Blackwell Publishing; 2007;152: 959–79. 10.1038/sj.bjp.0707471 17906685 PMC2078230

