# Supplementary figures and images for: Modified recipe to inhibit fruiting body formation for living fungal biomaterial manufacture
Source: PLoS One. 2019 May 13;14(5):e0209812. doi: 10.1371/journal.pone.0209812 (PMC6513072; doi:10.1371/journal.pone.0209812)

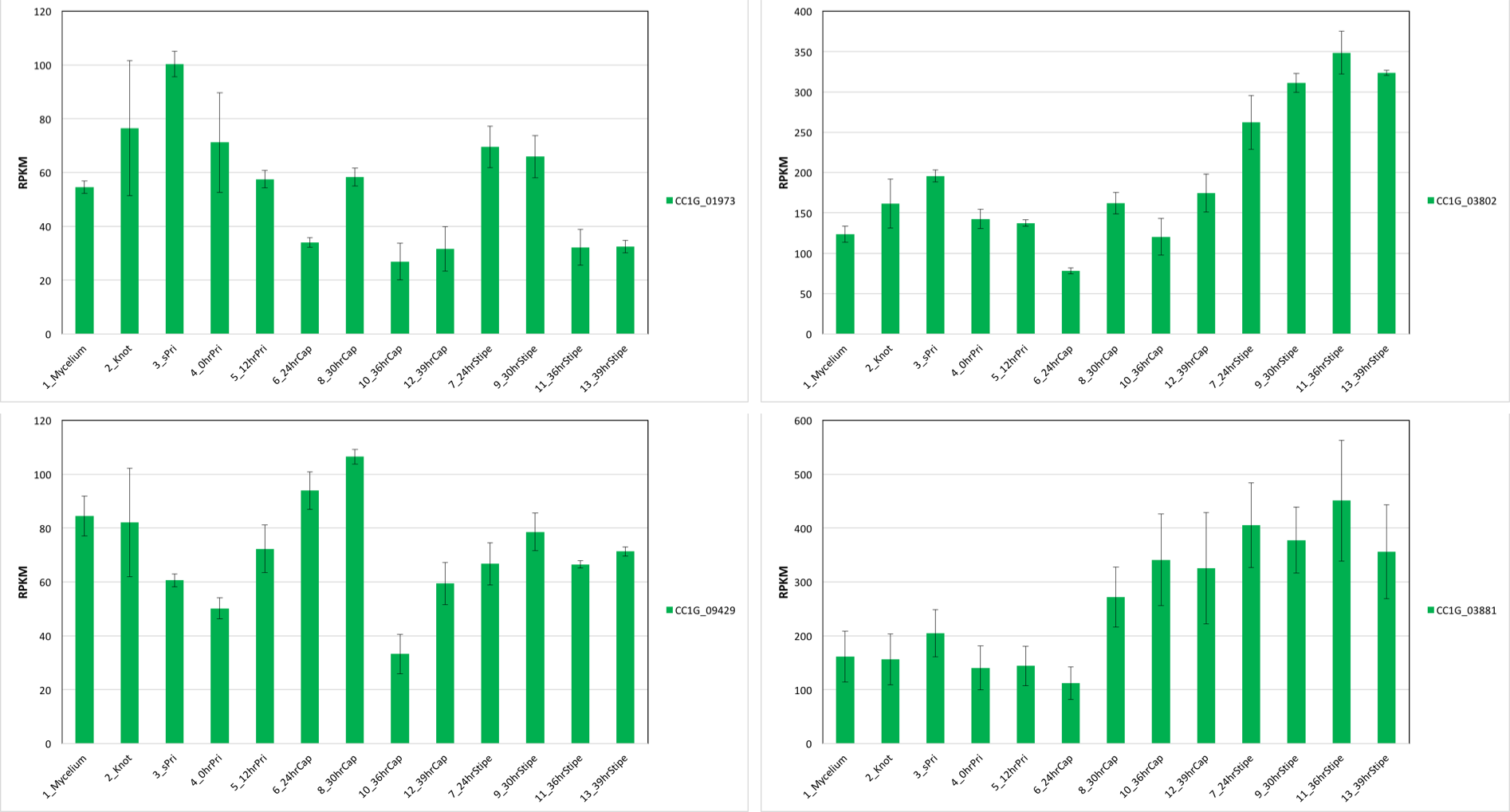

Supplement: S1 Fig — The values of expression levels were extracted from previous transcriptome study. (TIFF) [file pone.0209812.s001.tiff]
