# Supplementary material for: Modified recipe to inhibit fruiting body formation for living fungal biomaterial manufacture
Source: PLoS One. 2019 May 13;14(5):e0209812. doi: 10.1371/journal.pone.0209812 (PMC6513072; doi:10.1371/journal.pone.0209812)
Supplement: S1 Table — (PDF) [file pone.0209812.s002.pdf]

Table S1. Orthologues of GSK-3 substrates in *C. cinerea*

GeneID	Description	Functional Domains
**CC1G_00176T0**	ATP-dependent RNA helicase FAL1	Helicase_C;DEAD;
**CC1G_00238T0**	40s ribosomal protein s3ae-a	Ribosomal_S3Ae;
**CC1G_00303T0**	ribosomal protein L35	Ribosomal_L29;L29;
**CC1G_00346T0**	60s ribosomal protein l5-b	Ribosomal_L18p;
**CC1G_00637T0**	hypothetical protein	Ribosomal_L28e;
**CC1G_00659T0**	60s ribosomal protein l21-a	Ribosomal_L21e;
**CC1G_01103T0**	ubiquitin family proteint	HAD_IIID1;
**CC1G_01122T0**	60S ribosomal protein L7	Ribosomal_L30_N;Ribosomal_L30;L7;
**CC1G_01432T0**	vesicular-fusion protein sec18	AAA;CDC48_N;
**CC1G_01550T0**	hypothetical protein	-
**CC1G_01550T0**	hypothetical protein	-
**CC1G_01841T0**	SEC61	secE_euk_arch;SecE;
**CC1G_02496T0**	hypothetical protein	L15;
**CC1G_03278T0**	ATP synthase subunit 5	ATP_synt_delta;OSCP;
**CC1G_03510T0**	hypothetical protein	EMP24_GP25L;
**CC1G_03522T0**	histone H2A	Histone;CBFD_NFYB_HMF;
**CC1G_03575T0**	histone H2A	Histone;CBFD_NFYB_HMF;
**CC1G_03614T0**	lipase I	COesterase;
**CC1G_03615T0**	triacylglycerol lipase 3	COesterase;
**CC1G_03618T0**	lipase I	COesterase;
**CC1G_03630T0**	ribosomal protein L22	Ribosomal_L22e;
**CC1G_03667T0**	pre-mRNA splicing factor	LSM;
**CC1G_03726T0**	iron sulfur assembly protein 1	TIGR00049;Fe-S_biosyn;
**CC1G_03739T0**	40S ribosomal protein S29	Ribosomal_S14;
**CC1G_03856T0**	ubiquinol-cytochrome-c reductase complex subunit 6	UCR_14kD;

**CC1G_03881T0**	eukaryotic translation initiation factor 1	SUI1_MOF2;SUI1;
**CC1G_05155T0**	FACT complex subunit SPT16	SPT16;Rtt106;
**CC1G_05320T0**	g2/mitotic-specific cyclin cdc13	Cyclin_C;Cyclin_N;
**CC1G_05694T0**	60S ribosomal protein L30	Ribosomal_L7Ae;
**CC1G_06373T0**	AGC/YANK protein kinase	Pkinase;
**CC1G_06373T0**	AGC/YANK protein kinase	Pkinase;
**CC1G_06506T0**	Arg-6 protein	DUF619;Semialdhyde_dh;argC;AA_kinase;argB;
**CC1G_06565T0**	UDP-N-acetylglucosamine diphosphorylase	UDPGP;
**CC1G_06725T0**	60s ribosomal protein l15	Ribosomal_L15e;
**CC1G_06887T0**	hypothetical protein	-
**CC1G_07640T0**	histone H2A	Histone;CBFD_NFYB_HMF;
**CC1G_08167T0**	hypothetical protein	Cyt-b5;
**CC1G_08307T0**	fibrillarin-like protein	Fibrillarin;
**CC1G_08580T0**	lipase I	COesterase;
**CC1G_08662T0**	CAMK/CAMK1 protein kinase	Pkinase;
**CC1G_09116T0**	glyceraldehyde-3-phosphate dehydrogenase	Gp_dh_N;GAPDH-I;Gp_dh_C;
**CC1G_09313T0**	gamma-glutamyltranspeptidase	g_glut_trans;G_glu_transpept;
**CC1G_09342T0**	40S ribosomal protein S8	Ribosomal_S8e;S8e;
**CC1G_09348T0**	60S ribosomal protein L33	Ribosomal_L35Ae;
**CC1G_09429T0**	translation initiation factor eIF2 gamma subunit	eIF2_C;GTP_EFTU_D2;GTP_EFTU;
**CC1G_09504T0**	60s ribosomal protein l27	Ribosomal_L27e;
**CC1G_09696T0**	anaphase promoting complex subunit 1	-
**CC1G_09696T0**	anaphase promoting complex subunit 1	-
**CC1G_10540T0**	ribosomal protein L4/L1	Ribosomal_S9;
**CC1G_10719T0**	40S ribosomal protein S13	Ribosomal_S13_N;Ribosomal_S15;
**CC1G_10735T0**	cytochrome c	Cytochrom_C;
**CC1G_11064T0**	nascent polypeptide-associated complex subunit beta	NAC;
