# Supplementary material for: Modified recipe to inhibit fruiting body formation for living fungal biomaterial manufacture
Source: PLoS One. 2019 May 13;14(5):e0209812. doi: 10.1371/journal.pone.0209812 (PMC6513072; doi:10.1371/journal.pone.0209812)
Supplement: S2 Table — (PDF) [file pone.0209812.s003.pdf]

Table S2. Primer sequences for Real-time PCR

GeneID	Forward primer	Reverse primer
**CC1G_01973**	GCATGCAATCCGTGGAAGAC	ACACGGTTACGCTGGTTGAT
**CC1G_03802**	TGCGATCCCTCGCTTACATC	TCGCAGAGCTTCAAGACACC
**CC1G_03881**	AAACTCGAGCCCTCACCAAG	TGGTGAAGAAGAGGTGCGAC
**CC1G_09429**	TCCTGTGCGCGACTTTACTT	GAATCGAACCACCGGCTACA
**beta-tubulin**	GAGCAGTCCTGGTTGACCTC	TGTCCTTTGGCCCAGTTGTT
